# Infant feeding practices and determinant variables for early complementary feeding in the first 8 months of life: results from the Brazilian MAL-ED cohort site

**DOI:** 10.1017/S136898001800099X

**Published:** 2018-04-26

**Authors:** BLL Maciel, ML Moraes, AM Soares, IFS Cruz, MIR de Andrade, JQ Filho, FS Junior, PN Costa, CB Abreu, R Ambikapathi, RL Guerrant, LE Caulfield, AAM Lima

**Affiliations:** 1 Departamento de Nutrição, Saúde Coletiva e Nesc, Universidade Federal do Rio Grande do Norte, Campus Central, Av. Senador Salgado Filho 3000, Lagoa Nova, 59078-970 – Natal/RN, Brazil; 2 Department of Human Nutrition, Faculty of Medicine, Universidad Nacional de Colombia, Bogotá, Colombia; 3 INCT – Instituto de Biomedicina do Semiárido Brasileiro (IBISAB), Federal University of Ceará, Fortaleza, CE, Brazil; 4 Fogarty International Center, National Institutes of Health, Bethesda, MD, USA; 5 Department of Global Health and Population, Harvard T.H. Chan School of Public Health, Boston, MA, USA; 6 Center for Global Health, Division of Infectious Diseases and International Health, University of Virginia, Charlottesville, VA, USA; 7 Center for Human Nutrition, Department of International Health, The Johns Hopkins Bloomberg School of Public Health, Baltimore, MD, USA

**Keywords:** WHO core indicators, WAMI index, Dietary diversity, Minimum acceptable diet

## Abstract

**Objective:**

The present study aimed to describe breast-feeding, complementary feeding and determining factors for early complementary feeding from birth to 8 months of age in a typical Brazilian low-income urban community.

**Design:**

A birth cohort was conducted (*n* 233), with data collection twice weekly, allowing close observation of breast-feeding, complementary feeding introduction and description of the WHO core indicators on infant and young child feeding. Infant feeding practices were related to socio-economic status (SES), assessed by Water/sanitation, wealth measured by a set of eight Assets, Maternal education and monthly household Income (WAMI index). Two logistic regression models were constructed to evaluate risk factors associated with early complementary feeding.

**Results:**

Based on twice weekly follow-up, 65 % of the children received exclusive breast-feeding in the first month of life and 5 % in the sixth month. Complementary feeding was offered in the first month: 29 % of the children received water, 15 % infant formulas, 13 % other milks and 9·4 % grain-derived foods. At 6 months, dietary diversity and minimum acceptable diet were both 47 % and these increased to 69 % at 8 months. No breast-feeding within the first hour of birth was a risk factor for the early introduction of water (adjusted OR=4·68; 95 % CI 1·33, 16·47) and low WAMI index a risk factor for the early introduction of other milks (adjusted OR=0·00; 95 % CI 0·00, 0·02).

**Conclusions:**

Data suggest local policies should promote: (i) early breast-feeding initiation; (ii) SES, considering maternal education, income and household conditions; (iii) timely introduction of complementary feeding; and (iv) dietary diversity.

Infant feeding practices directly affect the nutritional status of children and consequently impact child survival^(^ ^1^ ^)^. Breast-feeding and complementary feeding practices have an important role in determining nutritional status, growth and development, imprinting physiological and metabolic mechanisms that lower the risk for infectious diseases^(^ ^2^ ^)^ and overweight/obesity-associated co-morbidities^(^ ^3^ ^)^.

Socio-economic status (SES) and demographic variables influence the individual nutritional changes occurring in the first years of life, as they may be determining variables for complementary feeding practices and continued breast-feeding^(^ ^4^ ^)^. Epidemiological changes due to the nutrition transition have been occurring worldwide in all population groups^(^ ^5^ ^)^.

In the last decades, the Brazilian context of intense political, economic and social transformation has greatly contributed to changes in the nutritional and epidemiological profile of the population^(^ ^6^ ^)^. The process of nutrition transition began more than 50 years ago and is still in progress. This has caused a paradoxical scenario where public health systems need to deal with the coexistence of both undernutrition-related infectious diseases and overweight/obesity co-morbidities^(^ ^7^ ^)^. In this context, national health, food and nutrition policies have changed.

The 2006 National Policy on Health Promotion (PNPS) and the 2011 National Policy of Basic Attention (PNAB) reinforced primary care and this has possibly promoted a closer follow-up of pregnant and lactating women. The National Food and Nutrition Policy (PNAN), first published in 1999 and updated in 2013, directs food and nutrition actions in order to promote overall healthy eating practices, prevention and control of nutritional disorders and stimulate intersectoral actions that promote universal access to food^(^ ^8^ ^)^. Beyond these actions, specific policies were created such as the National Strategy for the Promotion of Breastfeeding and Healthy Complementary Feeding in 2013 and the Stork Network in 2011^(^ ^9^ ^,^ ^10^ ^)^. These might have positively affected breast-feeding and infant feeding practices in Brazil.

The WHO recommends that infants should be exclusively breast-fed for the first 6 months of life for optimal growth, health and development^(^ ^11^ ^)^. The major negative aspects of early complementary feeding include an increased risk of infection, with consequent impaired growth and an increased risk for later obesity^(^ ^3^ ^,^ ^12^ ^)^. On the other hand, late and inadequate food introduction may lead to nutrient deficiencies^(^ ^13^ ^)^.

The WHO analysed breast-feeding duration in 108 UN countries and found that only 32 % of infants were exclusively breast-fed until 6 months of age^(^ ^14^ ^)^. One of the most debated determinants of breast-feeding duration and early complementary feeding is SES. In low- and some high-income countries, higher SES seems to be positively associated with breast-feeding duration^(^ ^15^ ^–^ ^18^ ^)^. This has not been extensively explored in poor communities of Northeastern Brazil in the last decade, deserving close attention.

Due to logistical limitations, few studies in infants allow close prospective observation of breast-feeding practices and timely food introduction. To evaluate early and late introduction of complementary feeding, cohort studies from birth to the first year of life would be more reliable. In addition, few studies use this approach to describe infant feeding practices in the context of the nutrition transition. This has made establishing risk factors for early complementary feeding difficult, which may comprise the development of specific policies. Thus, in the present study we describe breast-feeding, complementary feeding and determining factors for early complementary feeding from birth to 8 months of age in a typical Brazilian low-income urban community.

## Methods

### Study population, inclusion and exclusion criteria

The present study is part of a 5-year cohort multisite project administered by the Foundation for the National Institutes for Health and the Fogarty International Center called Etiology, Risk Factors and Interactions of Enteric Infections and Malnutrition and the Consequences for Child Health (MAL-ED)^(^ ^19^ ^)^.

The present study population is an urban low-income community located in Fortaleza, the capital of Ceará State, Northeast Brazil. The Parque Universitário community has approximately 33 000 people of predominantly low-class families, as previously described^(^ ^20^ ^)^.

The birth cohort study was initiated in November 2010 and followed up until February 2017. A total of 244 infants were enrolled within the first 17 d of life. Of these children, eleven moved or dropped out of the study (*n* 233). Inclusion criteria used for the study were: (i) birth weight greater than 1500 g; (ii) healthy newborn singleton child; (iii) child from a family intending to stay in the study area for the next 6-month period; (iv) no other child from the same family enrolled in the study; and (v) mother aged 16 years or above. Children were excluded from the study if they were premature, had congenital diseases, severe diseases that required hospitalization or any other condition that was severe or chronic, such as renal disease, chronic heart failure or severe liver disease^(^ ^19^ ^)^.

### Data collection

Field personnel were trained to collect data. At enrolment, mothers were asked about information on birth such as birth weight, time to breast-feeding initiation and if prelacteal feeding was given (any food except mother’s milk provided to a newborn before initiating breast-feeding). Data were collected twice weekly in household visits, from birth to 8 months of age of each child. Trained fieldworkers asked caregivers if, in the last 24 h, the child had consumed breast milk, animal milk, infant formula, water, tea, juice or any other liquids, solids or semi-solids.

WHO core indicators^(^ ^1^ ^)^ on breast-feeding were calculated using the weekly data. ‘Early initiation of breast-feeding’ was defined as the proportion of children who were put to the breast within an hour of birth. At each visit, breast-feeding was categorized as exclusive, partial or predominant, according to the WHO^(^ ^1^ ^)^ and Labbok and Starling^(^ ^21^ ^)^ definitions. ‘Exclusive breast-feeding’ was defined as breast-feeding with no other foods, water or other liquids given over the previous 24 h period, except drops or syrups containing vitamins, mineral supplements or medicine. ‘Predominant breast-feeding’ was defined as breast-feeding with the introduction of plain water or water-based liquids such as tea or juice. ‘Partial breast-feeding’ was considered when inclusion of other milks, formula and/or semi-solids was reported.

A food questionnaire was applied monthly to assess dietary diversity and quality; caregivers were asked if, in the last 24 h, the child had received grains, roots, meat, dairy products, yellow, orange or red fruits, dark green leafy vegetables and other fruits or vegetables. Complementary feeding core indicators^(^ ^1^ ^)^ were calculated using a combination of the weekly and monthly questionnaires, done referred to the last 24 h^(^ ^22^ ^)^. The ‘minimum acceptable diet’ was considered achieved by the infant if dietary diversity (≥4 different food groups) and meal frequency (≥2 meals/d for breast-fed infants and ≥4 meals/d for non-breast-fed infants) were accomplished, according to WHO definitions^(^ ^1^ ^)^.

A third questionnaire was used to assess SES. This questionnaire was adapted from the Demographic and Health Surveys^(^ ^23^ ^)^ and improved water and sanitization were based on WHO definitions^(^ ^1^ ^)^. The questionnaire was written in English, translated into Portuguese and back-translated for quality control. Demographic questions were on the child’s mother’s age, education and fertility history, as well as the education of the head of household. The SES section focused on water source and sanitation facilities, household assets, housing materials and ownership of land. Household monthly income in the local currency was also included in the questions.

From this questionnaire, we calculated the Water and sanitation, wealth measured by a set of eight Assets, Maternal education and monthly household Income index (i.e. WAMI index), described and validated by Psaki *et al*.^(^ ^24^ ^)^. Briefly, households with access to improved water or improved sanitation were assigned a score of 4 for each. Households without access to improved water or improved sanitation were assigned a score of 0 for each. These scores were summed. Eight priority assets were selected using random forest plots with height-for-age *Z*-score as the outcome: mattress, chair, table, television, refrigerator, bank account, kitchen and <2 people per room. For each asset, households were assigned a score of 1 if they had the asset and 0 if they did not have the asset. These scores were summed. Each child’s mother provided the number of years of schooling she had completed, ranging from 0 to 16 years. This number was divided by 2. Monthly household income was converted to US dollars using the exchange rate from 1 January 2010. This was divided into octiles using the following scores and cut-offs: 1 ($US 0–26·00), 2 ($US 26·01–47·00), 3 ($US 47·01–72·00), 4 ($US 72·01–106·00), 5 ($US 106·01–135·00), 6 ($US 135·01–200·00), 7 ($US 200·01–293·00), 8 (>$US 293·00). Thus, each of the components of the WAMI index were scored, ranging 0 to 8. Scores in water and sanitation, assets, mother’s education and income were summed and then divided by 32; the final WAMI index could range from 0 to 1^(^ ^24^ ^)^.

### Data analysis

Trained personnel double-entered data. Consistency checks and data cleaning were accomplished. We examined the distributions of continuous variables using the Kolmogorov–Smirnov test and presented variables that did not follow a normal distribution as median and interquartile range. Categorical variables were presented as frequency.

Since we observed that offering of water and other milks were the most common features of early complementary feeding in our sample, these two variables were considered outcomes investigated for risk factors. Independent variables included sex (male or female), birth weight, no breast-feeding within the first hour of birth (yes or no), maternal age, mother as the primary caregiver (yes or no), parity (if the mother was primiparous or not) and the WAMI index, since these could be variables influencing complementary feeding according to the literature^(^ ^1^ ^,^ ^14^ ^–^ ^17^ ^,^ ^19^ ^)^. All continuous variables were normalized before entering the models.

Initially, crude models were constructed in a bivariate analysis, exploring the effect of the variable alone in the outcomes with the crude OR and 95 % CI. Then, the variables were inserted into the adjusted model through the Enter Method and the final model adjustment was observed through the Omnibus tests of model coefficients, with *P*<0·05 considered significant. The Hosmer and Lemeshow test was also used, considering *P*>0·05 as reliable. Adjusted OR (AOR) and 95 % CI were shown to assess the risk found between a variable and the outcome analysed in the model. The analysis was performed using the statistical software package IBM SPSS Statistics version 23.

## Results

The percentage of infants who received colostrum and were born through caesarean delivery was high (98·6 and 55·7 %, respectively). Only 6·7 % of the studied infants received prelacteal feeding. Regarding maternal characteristics (Table 1), we observed that mothers were predominantly multipara, married and had more than 11 years of schooling. The WAMI index showed that the lowest scores were found for water/sanitization and maternal education (Table 1).

**Table 1:** Maternal, child and household general characteristics in the Brazilian MAL-ED cohort site

	Total (*n* 233)		
Variable	%	Median	IQR
Male child (%)	51·5	–	–
Age at enrolment (d)	–	9·0	7·0
Caesarean delivery (%)	55·7	–	–
Birth weight (g)	–	3400	0·6
Birth length (cm)	–	50·0	2·0
Colostrum feeding (%)	98·6	–	–
Prelacteal feeding (%)	6·7	–	–
No breast-feeding within the first hour of birth (%)	53·6	–	–
Mother as the primary caregiver (%)	93·2	–	–
Maternal age (years)	–	25·0	8·0
Maternal education (%)			
0–5 years	4·8	–	–
6–10 years	34·8	–	–
11–15 years	58·6	–	–
>15 years	1·9	–	–
Parity (%)		–	–
1	32·9	–	–
2–4	58·1	–	–
>4	9·0	–	–
Marital status			
Never married	8·1	–	–
Married	87·1	–	–
Divorced	3·3	–	–
Widowed	1·4	–	–
WAMI index variables*			
Water/sanitation	–	4·0	0·0
Assets	–	7·0	2·0
Maternal education	–	4·5	2·5
Income	–	7·0	1·0
Total	–	0·8	0·1

IQR, interquartile range.

* WAMI, Water, Assets, Mother’s education and Income index as a measure of socio-economic status described and validated by Psaki *et al.* ^(^ ^24^ ^)^. Each of the components of the index could range from 0 to 8, and the total index from 0 to 1. Water/sanitation: using WHO definitions of access to improved water and improved sanitation, households with access to improved water or improved sanitation were assigned a score of 4 for each. Households without access to improved water or improved sanitation were assigned a score of 0 for each. Assets: eight priority assets were selected using random forest plots with height-for-age *Z*-score as the outcome. For each asset, households were assigned a score of 1 if they had the asset, and 0 if they did not have the asset. Maternal education: each child’s mother provided the number of years of schooling she had completed, ranging from 0 to 16 years. This number was divided by 2. Income: monthly household income was converted to US dollars using the exchange rate from 1 January 2010. Income was divided into octiles using the following scores and cut-offs: ($US 0–26·00), 2 ($US 26·01–47·00), 3 ($US 47·01–72·00), 4 ($US 72·01–106·00), 5 ($US 106·01–135·00), 6 ($US 135·01–200·00), 7 ($US 200·01–293·00), 8 (>$US 293·00). Total: scores in water and sanitation, assets, mother’s education and income were summed and then divided by 32^(^ ^24^ ^)^.

**Fig. 1:**
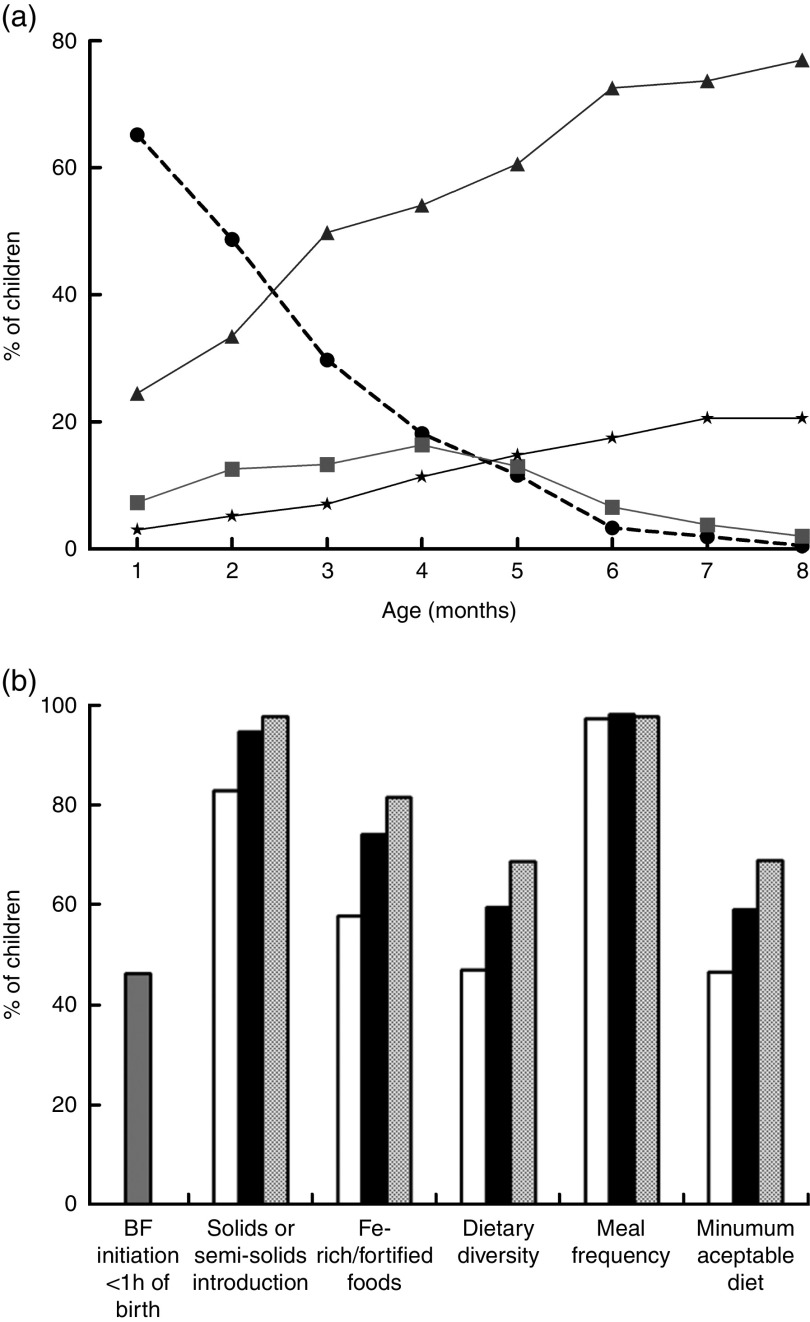
Breast-feeding patterns* and WHO core indicators on infant and young child feeding† in the Brazilian MAL-ED cohort site (*n* 233): (a) breast-feeding practices from 1 to 8 months of age (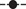, exclusive breast-feeding; 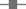, predominant breast-feeding; 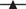, partial breast-feeding; 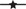, no breast-feeding); (b) breast-feeding (BF) initiation within 1 h of birth (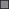), solids or semi-solids introduction, iron-rich/fortified foods, dietary diversity, meal frequency and minimum acceptable diet from 6 to 8 months of age (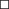, 6 months; 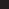, 7 months; 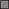, 8 months). *Exclusive breast-feeding: breast-feeding with no other foods or liquids given (not even water) over the previous 24 h period, except for drops or syrups containing vitamins, mineral supplements or medicine. Predominant breast-feeding: breast-feeding with the introduction of plain water or water-based liquids, such as tea or juice. Partial breast-feeding: breast-feeding with the inclusion of other milks, formula and/or semi-solids. †Dietary diversity: ≥4 different food groups. Meal frequency: ≥2 meals/d for breast-fed infants and ≥4 meals/d for non-breast-fed infants. Minimum acceptable diet: when dietary diversity and meal frequency are achieved

Infants exclusively breast-fed decreased from 65·2 % in the first month to 3·3 % by the sixth month of age, whereas the proportion of partially breast-fed infants increased significantly from 24·5 % in the first month to 72·6 % in the sixth month. Children receiving no breast milk also increased from 3·0 % in the first month to 20·6 % in the eighth month (Fig. 1(a)).

Of the children, 46·3 % were breast-fed within the first hour of birth (Fig. 1(b)). Concerning the other WHO core indicators for infant and young child feeding (IYCF), dietary diversity was met by only 47·1 % of children at 6 months of age and increased to 68·5 % at 8 months. Thus, minimum acceptable diet was met by only 46·6 % of children at 6 months and 68·8 % at 8 months (Fig. 1(b)).

**Fig. 2:**
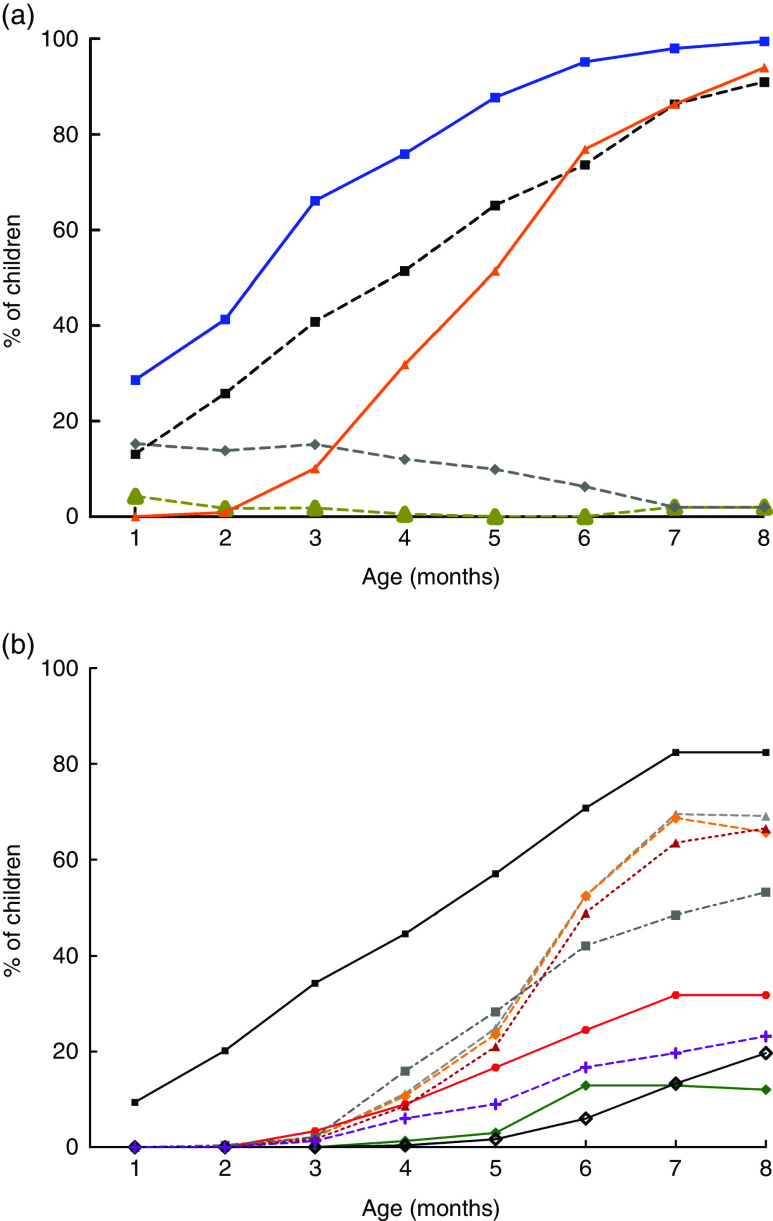
(colour online) Infant complementary feeding practices from 1 to 8 months in the Brazilian MAL-ED cohort site (*n* 233): (a) introduction of liquids (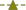, tea/coffee; 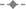, infant formula; 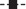, other milks; 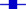, water; 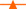, juice); (b) introduction of solids/semi-solids (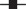, grains; 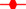, yellow, orange or red fruits; 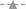, roots; 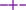, dairy products; 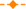, yellow or orange vegetables; 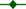, dark green leafy vegetables; 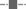, other fruits or vegetables; 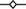, beans; 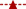, meat)

While a decline in exclusive breast-feeding occurred, increased provision of water and water-based preparations was observed, as shown in Fig. 2(a); and some solid and semi-solid foods, as shown in Fig. 2(b).

The frequency of liquids given to children changed over time (Fig. 2(a)). Water was the most common liquid given early during all months, with increasing frequency over time (28·7 % in the first month and 100 % in the eighth month). Infant formulas were mostly given in the first 3 months of life, with decreasing frequency thereafter. The same pattern was observed for tea and coffee: these liquids were more offered in the first 4 months of life, with almost no offering after this time. On the other hand, other animal milks were given early, from the first month (13·1 % of children), with increasing frequency over time (up to 91·0 % in the eighth month of life). The offering of juice also increased over the months (0·9 % in the second month *v.* 94 % in the eighth month).

Concerning solids and semi-solids, grains were the most offered foods to children. They were introduced early (9·5 % of the children in the first month) and their use increased over time to 82·4 % in the eighth month. Grains had a very similar offering as other milks (Figs 2(b) and 2(a), respectively). This can be explained by the fact that most of the mothers offering other milks did it as a combined preparation with wheat-, oat- and rice-based industrialized powder products (data not shown) and this was recorded as ‘grains’ in the study.

By the fourth month a larger variety of solid foods was given: grains (44·6 %), roots (11·2 %), meat (8·6 %), dairy products (6·0 %), yellow, orange or red fruits (9·0 %), other fruits or vegetables (15·9 %) and dark green leafy vegetables (1·3 %). These foods were offered more frequently from the fourth to the sixth month (Fig. 2(b)). It is interesting to note that by the fourth month the foods most often given were grains, other fruits or vegetables, and roots. By the sixth month, the foods most often given were grains (70·8 %), roots (52·4 %) and yellow/orange vegetables (52·4 %).

Comparing Figs 2(a) and 2(b), liquids were introduced earlier and given more often compared with solid foods. Actually, the only food offered similarly as liquids was grains, which showed similar percentages of feeding as other milks during the studied months.

Table 2 shows that no breast-feeding initiation within the first hour of birth was a risk factor for the introduction of water before 6 months of age (AOR=4·68; 95 % CI 1·33, 16·47). The WAMI index was inversely associated with the introduction of other milks before 6 months of age (AOR=0·00; 95 % CI 0·00, 0·02), with each increase of 0·01 in the WAMI index reducing the risk of early introduction of other milks by 100 %.

**Table 2:** Risk factors for the early introduction (<6 months of age) of water and other milks in the Brazilian MAL-ED cohort site (*n* 233)

	Early introduction of water				Early introduction of other milks			
Variable	OR	95 % CI	AOR	95 % CI	OR	95 % CI	AOR	95 % CI
Male child	1·27	0·59, 2,75	0·54	0·17, 1·73	1·40	0·81, 2·43	1·15	0·55, 2·43
Birth weight	1·28	0·58, 2·85	0·67	0·20, 2·31	1·04	0·59, 1·84	0·78	0·36, 1·69
No breast-feeding within the first hour of birth	1·67	0·80, 3,45	4·68	1·33, 16·47	1·25	0·77, 2·04	1·62	0·77, 3·39
Maternal age	0·96	0·88, 1·04	0·96	0·85, 1·08	0·99	0·94, 1·05	1·00	0·93, 1·09
Mother as the primary caregiver	0·51	0·06, 4·09	1·13	0·13, 10·26	0·75	0·23, 2·46	1·86	0·47, 7·42
Primiparous	4·34	1·27, 14·81	1·42	0·30, 6·59	0·82	0·45, 1·48	1·13	0·44, 2·89
WAMI index*	0·015	0·00, 8,97	0·01	0·00, 14·99	0·00	0·00, 0·02	0·00	0·00, 0·02

AOR, adjusted OR, considering all variables exposed in the model.

* WAMI, Water, Assets, Mother’s education and Income index as a measure of socio-economic status described and validated by Psaki *et al.* ^(^ ^24^ ^)^.

## Discussion

Studies have shown that exclusive breast-feeding and timely safe introduction of complementary foods are key determinants of health, cognitive development, and both present and future nutritional status^(^ ^1^ ^,^ ^25^ ^–^ ^28^ ^)^. Many developing countries face epidemiological changes due to nutrition transition^(^ ^5^ ^)^, but few studies have investigated breast-feeding and complementary feeding practices in the light of this scenario. In the present study, we used a close observation cohort design that allowed for the description of breast-feeding and infant feeding practices using the WHO core indicators for IYCF. Our results allowed the observation of both early and late complementary feeding practices since we collected and analysed data from 0 to 8 months of age. Moreover, to our knowledge, the present study is the first to elucidate determining key factors for early complementary feeding using the WAMI index as a measure of SES.

Although Brazilian national surveys from 1986 to 2008 showed an increase in the frequency of exclusive breast-feeding at 6 months from 3·1 to 41·0 %^(^ ^29^ ^)^, in our study only 3·3 % of children were exclusively breast-fed at 6 months of age. This probably reflects different methodological procedures to assess exclusive breast-feeding. National surveys commonly use a cross-sectional design, obtaining information on the proportion of infants exclusively breast-fed on the prior day. In the present study, we followed children prospectively and frequent interruptions and recaptures in exclusive breast-feeding were detected, as previously discussed^(^ ^30^ ^)^. In our study, the first interruption of exclusive breast-feeding led to the classification of the child in a breast-feeding pattern other than exclusive and the correct timing of this was possible only considering the multiple visits planned in our study design. Nevertheless, data presented here are in accordance with our previous studies with similar populations^(^ ^31^ ^–^ ^33^ ^)^, demonstrating the constant need for stronger health policies on breast-feeding promotion in the study area.

Some factors such as the offering of prelacteal feeding, non-provision of colostrum and the provision of liquids and semi-solids can compromise the practice of breast-feeding^(^ ^13^ ^)^. Studies have also demonstrated that a high incidence of prelacteal feeding is associated with low colostrum feeding^(^ ^34^ ^,^ ^35^ ^)^. This was not observed in our study, since we found low prelacteal and high colostrum feeding. Exclusive breast-feeding has been positively associated with paternal support for breast-feeding and negatively associated with delivery by caesarean section^(^ ^34^ ^)^ and primiparity^(^ ^36^ ^)^. In the present study, more than half of infants (55·7 %) were born by caesarean delivery and this may have affected breast-feeding success^(^ ^1^ ^,^ ^37^ ^)^.

Our overall results show that the provision of liquids and solid/semi-solid foods may have compromised exclusive breast-feeding. Liquids were given earlier and more often compared with solid/semi-solid foods. The offering of different kinds of liquids, such as tea/coffee, juice and especially water, which was given early and frequently, likely derives from the mothers’ misperception that the child may dehydrate if only breast milk is offered in this tropical region with continuous high temperatures during the year. Numerous studies have demonstrated that healthy breast-fed babies living in hot climates do not need water in addition to breast milk^(^ ^38^ ^,^ ^39^ ^)^. Additionally, compared with breast milk these liquids are nutrient-poor, may be contaminated with enteropathogens and their consumption is associated with a higher risk of morbidity and mortality in the first 6 months of life^(^ ^40^ ^)^.

In the present study, we found no significant statistical associations between SES and exclusive breast-feeding in the first 6 months of life (data not shown), likely because the number of infants exclusively breast-fed by 6 months of age was small. Nevertheless, water and other milks were the most common liquids offered early to children. This led us to investigate determining factors for these practices. Interestingly, breast-feeding initiation after an hour of birth was a determining factor for the early introduction of water. The WHO has systematically focused on recommending early initiation of breast-feeding^(^ ^1^ ^,^ ^41^ ^,^ ^42^ ^)^. This has been positively associated with exclusive breast-feeding success^(^ ^37^ ^)^ and our data confirm these findings.

It is interesting to note that the offering of infant formulas decreased over time, while that of other milks increased (Fig. 2(a)). This possibly reflects the mothers’ access to infant formulas, but that these products might be too expensive to maintain in the long term.

Our logistic regression model, showing that increased WAMI index was inversely associated with the early provision of other milks, is an indication that the use of a complex SES indicator is important to assess determining infant feeding practices. The WAMI index is a composite index for SES evaluation that takes account of income, wealth and maternal education. Other strengths of using the WAMI index in the present study are that it was developed in the same population, has proved to be applicable for comparisons between seven other country sites in South Asia, sub-Saharan Africa and Latin America, and is strongly associated with height-for-age *Z*-score. These WAMI index characteristics provide evidence of the importance of using a robust measure of household SES, rather than single measures of wealth or education. Although using this combined indicator will not enable us to assess single components of the WAMI score as risk factors, we do not believe SES could be truly improved if only single components are targeted or assessed because, conceptually, SES is combined measure^(^ ^24^ ^)^. Thus, public policies should not address only single components of SES, but a framework of variables important for community development.

Moreover, our result showing that a higher WAMI index might be protective against the early introduction of other milks is particularly interesting. This might reflect that even though Brazil has been facing strong socio-economic changes in the last two decades, with improved population income and access to assets, higher SES is still a positive determining factor for early complementary feeding.

Our results showed that early complementary feeding was common and not only other milks were introduced early, but also fruits and vegetables, roots and meat from 4 months of age. Data indicating early provision of complementary feeding are in accordance with other studies showing that early introduction of liquids and semi-solids is a common practice in Brazil^(^ ^43^ ^–^ ^45^ ^)^. Infants who are partially breast-fed during the first 6 months of life experience more morbidity from gastrointestinal infection and consequent undernutrition than those who are exclusively breast-fed^(^ ^13^ ^)^. This may be the case for our study children, and further analysis should focus on that hypothesis since partial breast-feeding was the most common breast-feeding pattern observed.

Moreover, early introduction of complementary feeding can cause malnutrition, by nutrient deficiencies or excesses, when the foods offered do not satisfy nutritional requirements^(^ ^40^ ^)^. The most consumed foods during the six study months were grains and roots, both of which are rich in carbohydrates. Interestingly, grains were offered similarly with other milks during the studied months.

A study from all Brazilian capitals and the Federal District has shown that a high proportion of children receive porridge, prepared with grains and other milks^(^ ^46^ ^)^. This is likely the case for the children from the present study. The most consumed grains by our population were maize and infant industrialized preparations composed of maize, rice, wheat, sugar and mixed minerals. This has a plausible impact on nutrient adequacies and nutritional status, and further studies should focus on these issues.

Few recent studies have been published on IYCF practices in poor Northeastern Brazilian communities, but recent Brazilian^(^ ^38^ ^)^ and global data indicate that increasing energy density is not related to an increase in the micronutrient content. In fact, the micronutrient content of preparations such as porridge may not meet the child’s requirements and much of the recent scientific debate on exclusive breast-feeding duration has centred on micronutrient adequacy^(^ ^13^ ^)^.

In reflection of some national policies such as the National Strategy for Healthy Complementary Feeding (ENPACS) in improving complementary feeding in the first year of life^(^ ^47^ ^)^, Brazilian time trends in complementary feeding introduction and quality of infant feeding practices seem to have improved over the last decade^(^ ^45^ ^,^ ^48^ ^)^. Nevertheless, our data might indicate that for specific vulnerable communities timely and adequate complementary feeding introduction can still be improved.

Concerning the WHO core indicators for complementary feeding, iron-rich/fortified foods were introduced later, and by 8 months not all children had been introduced to these foods. In the present study, however, meat is not a highly consumed dietary component. By the sixth month, only 48·9 % of the infants received meat. The consumption of dark green leafy vegetables was low (12·9 %) and few children received beans (6·0 %). Organ meat and eggs were also infrequently consumed (2·2 %; data not shown). Although meal frequency was adequate, dietary diversity did not meet recommendations, which led to almost half of the children not meeting the minimum acceptable diet by 6 months of age. The adherence to this indicator increased by 8 months, but still a considerable number of children did not meet recommendations. Possible causes for these results might have been the low offering of foods listed above and policies should focus on increasing access for mothers to provide nutrient-dense foods. The low offer of different groups of foods, leading to low dietary diversity, could be explained by two factors, which should be explored in further studies: (i) little/compromised access; and/or (ii) lack of knowledge that all these foods could be offered in complementary feeding.

Our findings on the WHO core indicators for IYCF are not easily comparable to those from the literature, given that the few Brazilian studies on this topic use different indicators and do not present time trends considering multiple visits, as done in the present study. Thus, efforts must be taken to study IYCF practices using the WHO core indicators since these have been proved to be easily measured, reliable and associated with linear growth^(^ ^49^ ^,^ ^50^ ^)^. Studies in low-income countries have found similar proportions of dietary diversity and minimum acceptable diet^(^ ^49^ ^,^ ^51^ ^)^, showing that strategies to understand mothers’ decision-making process for infant feeding practices and access to foods are needed worldwide.

One of the limitations of our results is that data are not nationally representative. Nevertheless, compared with the other MAL-ED sites, the Brazilian site presents a different population when considering SES^(^ ^24^ ^)^, which makes it interesting to explore individually for IYCF practices, especially for local policy strategies. It is likely that the infant feeding practices reported here reflect those of the majority of poor urban communities, known as *favelas*, since our study community is a very typical north-eastern *favela*, as previously described^(^ ^20^ ^)^. Moreover, to our knowledge, the present study is the first Brazilian one to observe infant feeding practices prospectively, using twice weekly data collection, allowing for very strict classification of breast-feeding patterns and infant feeding practices, considering the WHO core indicators for IYCF. Thus, we suggest that larger national studies using similar data collection and analysis models are needed.

In summary, although almost all infants in our study were breast-fed, the proportion of exclusive breast-feeding up to 6 months was very low. Liquids, especially water and other milks, and solids/semi-solids were introduced to infants early in the first month of life. Grains were the group of solid foods mostly offered, while the inclusion of beans and dark green leafy vegetables was low, leading to low minimum acceptable diet adherence at 6 months. Other milks were used frequently, possibly in combination with grains in micronutrient-deficient meals. Breast-feeding initiation within the first hour of birth and higher SES were determinant variables preventing the early introduction of water and other milks, respectively. This leads us to conclude that local policies are needed to improve exclusive breast-feeding duration, prevent the early introduction of water and the start of complementary feeding, and promote better complementary feeding practices. These policies should include strategies to promote: (i) early breast-feeding initiation; (ii) SES, considering maternal education, income and household conditions; (iii) timely introduction of complementary feeding; and (iv) dietary diversity in complementary feeding.
